# A personal account of contributions to equity, diversity and inclusion within the Canadian chemistry community

**DOI:** 10.1038/s42004-023-00820-w

**Published:** 2023-01-26

**Authors:** Hind A. Al-Abadleh

**Affiliations:** Department of Chemistry and Biochemistry, Wilfrid Laurier University, Waterloo, ON N2L 3C5 Canada

**Keywords:** Atmospheric chemistry, Communicating chemistry

## Abstract

Efforts to address equitable access to education and research in chemistry are ongoing in many countries around the world. Here, Professor Hind A. Al-Abadleh provides a personal account of her contributions to equity, diversity, and inclusion in the Canadian chemistry community.

## Teaser

**Fig. 1:**
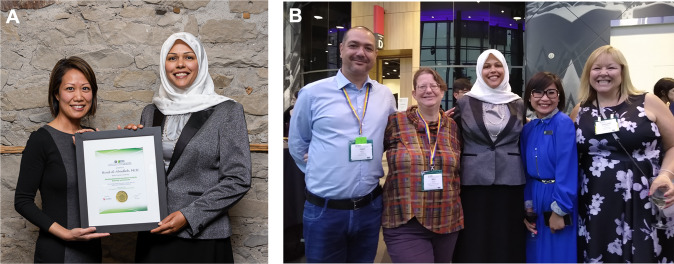
Selected photos from the awards reception at the 2022 Canadian Chemistry Conference and Exhibition. Professor Hind Al-Abadleh with (**A**) Dr. Erica Tiong (Gilead Sciences) who presented the award, and (**B**) Dr. Tiago Vieira (Gilead Sciences), Professor Nola Etkin (University of Prince Edward Island and co-founding member of WIDE), Dr. Josephine Tsang (Executive Director, CIC), and Professor Stephanie MacQuarrie (Cape Breton University and Chair of WIDE). The author affirms that informed consent for publication of the images was obtained from the identifiable individuals.

On June 14, 2022, at the Canadian Chemistry Conference and Exhibition in Calgary, Alberta, I delivered the award lecture for the inaugural Canadian Society of Chemistry (CSC) Gilead Award for Excellence in Equity, Diversity, and Inclusion. The lecture was scheduled in a special symposium entitled, “Affecting Institutional Equity, Diversity, and Inclusion (EDI) Mindsets”, organized by Professor Stephanie MacQuarrie, Cape Breton University, Dr. Nimrat Obhi, Beyond Benign, Professor Dipesh Prema, Thompson Rivers University, Dr. Janelle Sauvageau, National Research Council, Dr. Bryony McAllister, Transport Canada, Professor Nola Etkin, University of Prince Edward Island, and Professor Jean-Denys Hamel, University of Lethbridge. Fig. 1 shows photos from the award reception with representatives from Gilead Sciences, the Chemical Institute of Canada (CIC), and the CSC Working towards Inclusion, Diversity, and Equity (WIDE) working group, then committee. This paper is an excerpt of the award lecture with a focus on my 10+ years of contributions to EDI within the Canadian Chemistry Community.

## Motivation for engagement in EDI work

My academic career started in 2005 as a tenure-track Assistant Professor in the Department of Chemistry and Biochemistry at Wilfrid Laurier University in Waterloo, Ontario, Canada. My interest in chemistry started in high school when I was introduced to environmental pollution of all kinds. Upon studying chemistry at the university level, I knew that through chemistry, we can fix climate change and clean the environment. Over the years, I noticed that the vast majority of students and fellow scientists I met were attracted to pursuing education and careers in chemistry to solve societal and environmental challenges, and it seemed to me that fewer than 1% originally pursued chemistry to fix the gender and race gap in this scientific field^1,2^. But here we are, in 2023, and some of us who started in chemistry with a genuine spark of curiosity come face-to-face with a culture in the scientific chemistry community that is not as open and welcoming to diverse voices and backgrounds as we need as a society given the scale of the challenges on the ground. Our modern human civilization is dealing with an existential crisis in the form of global climate change because we built that civilization at the expense of the whole planet and every living species. As trained scientists and problem solvers, we have a lot to offer our human family in terms of knowledge and expertise to chart a way out of the environmental, social, and economic mess we are in.

My desire to engage in EDI work as an academic scientist was motivated by personal encounters throughout my education and career. My first motivator was the history of the environmental movement since the industrial revolution. As an environmental chemist who works in Canada and is always asked what we can do to solve environmental problems, I am inspired by the life and work of Dr. David Suzuki, a Canadian academic, science broadcaster, and environmental activist. He emphasizes the need for an attitude shift in the current economic system, which equals the importance of society, economy and the environment, to a new system, where nature dictates the limits of human activities and economic models. When I had the privilege of meeting Professor Faisal Moola, who worked very closely with Suzuki, he emphasized the importance of engaging ethnic communities in Canada in the environmental movement to ensure the success of initiatives aimed at changing behaviors at the individual and community levels. The second motivator for my EDI work was my lived experiences: growing up in all-girls public schools in the United Arab Emirates, which prepared me exceptionally well to enter the chemistry program at the university level empowered and motivated; my mixed experiences as an international PhD student and postdoc in the United States; and later my mixed experiences as an immigrant to Canada through the skilled worker federal immigration program. I often get asked if my identities as a visibly Muslim female and Arab affect my reception and inclusion among other scientists. The answer I give is that engaging with scientists keeps fueling my desire for making transformative change in the ‘culture’ of science and the public perception of ‘what scientists look like’. This change needs to start early, has to be reinforced at different educational and career stages, and demands the recruitment, advancement, and engagement of scientists from marginalized groups with the academic and wider communities.

## Mentorship style of chemistry research students

Through research projects in environmental chemistry and professional leadership roles, I consciously aim to increase awareness, create opportunities, and remove barriers. The research activities in my lab aim to directly address the United Nations Sustainable Development Goals on climate, water, health, education, and partnerships, and indirectly the goals on gender equality, reduced inequalities, sustainable cities and communities. Most science students interested in joining my research group want to make a difference in the world they live in by engaging in environmental chemistry research. These students come with their own life experiences, career aspirations, family expectations, strengths and weakness, likes and dislikes, outlook on life, and intellectual and physical abilities. Past and current undergraduate and graduate students in my lab became first-time authors on scientific papers, presenters at conferences, and recipients of awards and scholarships. Reaching that level of producing scientific knowledge and competing for recognition required from the students the willingness to learn and adoption of a growth mindset. It required from me as the research mentor creating a customized training plan that appreciates the individuality of each student, builds on each student’s strengths, gives clear direction to improve their weaknesses, and equips them with transferrable technical and communication skills that support their own life and career goals. It also required from me creating respectful group dynamics that encourage confidence building through engaging in critical thinking, scientific ideas exchange, and mentorship of junior students. In addition, research mentorship also meant allying with students in my research group when facing unique challenges because of their abilities or identities upon navigating the academic and job market systems. To sum it up, retaining and advancing students in science in general, and chemistry in particular, require investing time and energy in a customized, respectful, and productive mentor–mentee relationship.

## Affecting change at organizational levels

Advancing the EDI efforts as a leader through professional service roles takes other approaches because the challenges are qualitatively different from those in managing a research group. For example, several reports on the status of women and other minoritized groups in science, engineering, technology and math showed that their scientific scholarly output is under-cited and under-valued in the form of awards and other recognitions^3–5^. My guiding EDI action principles are fueled by my own lived experiences, aim to go beyond being performative, and are informed by the social science and psychology research needed to transform culture. Since most academic and professional scientific committees and groups tend to have hierarchal structures, I was selective with the groups I joined and strived to be engaged with action-oriented leaders who are willing to learn, apologize, sponsor, and amplify. The groups I joined where my advocacy efforts led to tangible outcomes include WIDE, CIC Environment Division, Nano Ontario Inc., and Atmosphere-Related Research in Canadian Universities (ARRCU), a special interests group with the Canadian Meteorological and Oceanographic Society.

As a member of WIDE from 2016–2021, I advocated for and co-analyzed the invited speaker gender diversity at the symposia of the CSC meetings held between 2016–2018, drafted the Conference Code of Conduct Policy for the 2018 CSC national meeting, and advocated for hosting a Presidential Event on EDI at the 2018 CSC national meeting in Edmonton, which had the title, “Catalyzing Change – Diversity in Canadian Chemistry”. I also advocated for including ‘equity’ in the CSC Accreditation Guidelines for undergraduate chemistry programs, contributed to the development of the self-identification policy and questionnaire, which was implemented in the 2021 virtual national meeting for the first time, and advocated for sending open calls for the recruitment of new members to the boards of the CIC, CSC, WIDE, and subject division executives.

As the Chair of the CIC Environment Division from 2019–2022, I launched the CIC Environment Division Early Career Award (2020), which I proposed and advocated for as Vice Chair to the Division Executives, took steps to increase the division membership and diversity through widely distributing membership posters, sponsoring student-led conferences, and sending open call emails for recruitment to the division executives from all career stages and professions (academia, industry, government). I also launched the photoshoot competition that aims to highlight the diversity of ideas and teams in environmental chemistry, chemical engineering and technology in Canada.

I joined Nano Ontario Inc. in 2012 as a Board Member representing Wilfrid Laurier University. Nano Ontario Inc. is a not‐for‐profit organization that aims at promoting the interests of nanoscale science in Ontario. I wrote the diversity statement and proposed to the board the establishment of the awards program for women in nanoscience and nanotechnology in Ontario. This proposal passed unanimously, and I became the Chair of the awards program, which to date, presented 18 awards to early- and mid-career and lifetime achievements in nanoscience. This program was expanded recently to include postdoctoral scholars and research associates. We also changed the eligibility criteria for the nominations to include all minoritized groups as defined by the Natural Sciences and Engineering Research Council of Canada. I also advocated for Nano Ontario Inc. to sign up for the 50‐30 Government of Canada Challenge to increase the diversity of the board for achieving gender parity (50% women and/or non-binary people) and significant representation (30%) of members of other equity-deserving groups.

As an atmospheric chemist, I was recruited to join ARRCU in 2017, which started as a working group and later became a special interests group with Canadian Meteorological and Oceanographic Society. As a member, then Vice-Chair, I contributed to drafting the Terms of Reference and White Paper to emphasize education, partnerships and outreach, and drafted an open call email for recruitment of new members on the executive and advisory boards. Inspired by the recommendations of the 2050 Climate Science report from Environment and Climate Change Canada^6^, I organized a virtual knowledge mobilization workshop that gathered a diverse group of speakers from government and academic scientists, policy researchers, scientific journal editors, environmental lawyers and journalists. I recruited an up-and-coming science communicator to co-host this workshop with me. As the current Chair of ARRCU, I organized the second knowledge mobilization workshop on advancing the United Nations Sustainable Development Goals on ocean and the climate with emphasis on the indigenous knowledge and community outreach and engagement. My goals as Chair are to amplify the scientific output and voices of all the members to the media, policy makers and general public, support their ideas, and facilitate connections at different levels through online and in-person events.

## Reflections and final thoughts

Overall, the voluntary initiatives I mentioned above with WIDE, CIC Environment Division, Nano Ontario Inc., and ARRCU worked because the past and current leaders of these groups and organizations embodied the qualities of inclusive leaders. Bourke and Dillon of Deloitte list these qualities to be curiosity, cognizance of bias, commitment, collaboration, courage and cultural intelligence. To affect organizational EDI mindsets, the efforts should start with tenured faculty and relatively established scientists who are part of the scientific community and know it inside out. Because there is no cookie-cutter model to EDI initiatives that would work everywhere with the same degree of effectiveness, I invite you to customize your action plans to have a maximum and lasting positive cultural impact on the members of your research and professional scientific groups.
